# Effect of Thermal Buoyancy on Fluid Flow and Residence-Time Distribution in a Single-Strand Tundish

**DOI:** 10.3390/ma14081906

**Published:** 2021-04-11

**Authors:** Dong-Yuan Sheng, Pär G. Jönsson

**Affiliations:** 1 Department of Materials Science and Engineering, Royal Institute of Technology, 10044 Stockholm, Sweden; parj@kth.se; 2 Westinghouse Electric Sweden AB, 72163 Västerås, Sweden

**Keywords:** thermal buoyancy, computational fluid dynamics (CFD), residence-time distribution (RTD), fluid flow, heat transfer, mixing

## Abstract

Natural convection of molten steel flow in a tundish occurs due to the temperature variation of the inlet stream and heat losses through top surface and refractory walls. A computational fluid dynamics (CFD) model was applied to study the effect of thermal buoyancy on fluid flow and residence-time distribution in a single-strand tundish. The CFD model was first validated with the experimental data from a non-isothermal water model and then applied to both scale-down model and prototype. The effects of flow control devices, including weir, dam and turbulence inhibitor, were compared and analyzed. Parameter studies of different heat losses through the top surface were performed. The results show that thermal buoyancy has a significant impact on the flow pattern and temperature distributions of molten steel in the tundish. The increase of heat loss through the top surface shortens the mean residence time of molten steel in the tundish, leading to an increase in dead volume fraction and a decrease in plug flow volume fraction.

## 1. Introduction

A tundish, working as a buffer and distributor of liquid steel between the ladle and continuous casting molds, plays a key role in affecting the performance of casting and solidification, as well as the quality of final products, referred to as “Tundish Metallurgy” [1]. Considerable research efforts have been made in academia and industry over many decades to fully exploit and enhance the metallurgical performance of the tundish [2,3,4,5]. The work on optimizing tundish design and operation parameters to meet the demand of improvement in steel quality is one of important research projects for metallurgists. 

During a continuous casting process, the hotter liquid steel will be poured from the ladle into the tundish. The temperature of the inlet stream from the ladles may vary from different heats, or on the ladle’s teeming time. In addition, there are conductive heat losses through the wall and radiative heat loss through the bath surface. This leads to a variation of steel temperature in the tundish, which cannot be neglected in both mathematical simulation and practical operation.

Large numbers of studies on mathematical models and water models have been reported on the analyses of fluid flow and heat transfer in tundish, with focus on the optimization of flow control device. A summary of the previous modelling works of tundish under non-isothermal conditions can be found in Table 1. These studies led to considerable improvements in understanding the various flow phenomena associated with tundish performance and operations. Nonetheless, there are far fewer publications on non-isothermal results compared to publications on isothermal results. Within the published non-isothermal studies, many researchers focused on a unitary thermal boundary condition, either varying the inlet temperature or the heat losses to surrounding, changing the liquid temperature in a tundish. Very few performed studies considering a systematic variation of thermal status of molten steel in a tundish. Under real production conditions, heat loss during casting can be complex and may involve both temperature difference of the incoming liquids and heat loss, through tundish boundaries. The heat flux of boundary condition is not always constant, as most studies assumed (shown in column of “Heat flux” in Table 1). Often, the heat flux can be changed according to the working conditions of refractory lining materials. Detailed quantitative analysis of the thermal buoyancy effect on flow patterns and residence-time distribution, considering all the above mentioned thermal boundary conditions, has rarely been reported in previous publications.

In the present study, a transient computational fluid dynamics (CFD) model has been applied to calculate a non-isothermal water model with a focus on the temperature variation of the inlet stream. The CFD model was validated with the measured temperature in the water model. The developed CFD model has also been used for the simulation of a prototype in order to investigate the effect of thermal buoyancy caused by the heat losses to the surrounding while keeping the inlet temperature unchanged. Two different tundish configurations of flow control devices were studied: (i) weir and dam and (ii) weir, dam and turbulence inhibitor. Parameter studies of different heat losses through the top surface were performed. Residence-time distribution (RTD) of fluid was selected as an index of the tundish performance. Two categories of RTD (E-curve and F-curve) were analyzed. The flow fields, temperature distributions and RTD curves aimed at achieving optimum control of flow and temperature of the molten steel in the tundish. The long-term objective of this work is to develop a mathematical model that can simulate the fluid flow and heat transfer in a tundish during an entire ladle changeover operation, under transient thermal boundary conditions. Herein, the research work in this paper was confined to describe the results of the first-step study on modelling development. 

## 2. Model Description

### 2.1. Mathematical Modelling

CFD software STAR-CCM + V.13 (Siemens PLM software, Plano, TX, USA) was used to model fluid flow, heat transfer and residence-time distribution in the tundish [28]. The assumptions made for the mathematical model are described below:

- The model is based on a 3D standard set of the Navier–Stokes equations [29].
- Non-isothermal and transient flow is calculated for the water model.
- Non-isothermal and steady-state flow is calculated for the prototype.
- The realizable *k*-*ε* model is used to describe the turbulence [30].
- Boussinesq model is applied to calculate the natural convection flow.
- The heat losses of water model are ignored. The heat losses of prototype are considered.
- The free surface is flat and is kept at a fixed level. The slag layer is not included.

#### 2.1.1. Transport Equation 

Equations (1)–(3) are used to describe the continuous phase.

Continuity:

(1) $$\frac{\partial \left(\rho u_{j}\right)}{\partial x_{j}}=0$$

Momentum:

(2) $$\rho u_{j}\frac{\partial u_{i}}{\partial x_{j}}=-\frac{\partial P}{\partial x_{i}}+\frac{\partial }{\partial x_{j}}\left[\left(\mu +\mu_{t}\right)\left\{\frac{\partial u_{i}}{\partial x_{j}}+\frac{\partial u_{j}}{\partial x_{i}}\right\}\right]+g_{i}\left(\rho -\rho_{0}\right)$$

Thermal energy:

(3) $$\rho C_{p}\frac{\partial T}{\partial t}+\rho C_{p}\frac{\partial \left(u_{j}T\right)}{\partial x_{j}}=\frac{\partial }{\partial x_{j}}\left[\left(k_{0}+\frac{C_{p}\mu_{t}}{Pr_{t}}\right)\frac{\partial T}{\partial x_{j}}\right]+S_{T}$$

where *ρ* is the density; *C_p_* is the heat capacity; *µ_t_* is the turbulent viscosity; *Pr_t_* is the turbulent Prandtl number (the value of 0.9). *S_T_* represents the source term of energy equation. 

Two passive scalar equations are solved in the CFD model: (i) an instantaneous addition of the tracer at the inlet (E-curve); (ii) a continuous addition of tracers at inlet (F-curve). The passive scalar transport equations are solved at each time step once the fluid field is calculated.

(4) $$\rho \frac{\partial \bar{C}}{\partial t}+\rho \bar{u_{j}}\frac{\partial \bar{C}}{\partial x_{j}}-\frac{\partial }{\partial x_{j}}\left[\rho D_{eff}\frac{\partial \bar{C}}{\partial x_{j}}\right]=0$$

where *D_eff_* is the effective diffusivity. The velocity field is solved and obtained from a steady-state simulation and remained constant during the calculation of the passive scalar.

#### 2.1.2. Analysis of RTD Curves

E-curve can be plotted based on the dimensionless outlet concentration (C-curve). Actual mean residence time is presented in Equation (5) [31,32].

(5) $$\bar{\tau }=\frac{\int_{0}^{\infty }tC\left(t\right)dt}{\int_{0}^{\infty }C\left(t\right)dt}$$

The plug flow volume fraction (*V*_p_*/V*), mixed flow volume fraction (*V*_m_*/V)* and dead volume fraction (*V*_d_*/V*) were calculated through Equations (6)–(8) [33]. 

Dead volume fraction,

(6) $$V_{d}/V=1-\frac{\bar{\tau }}{\tau }$$

Plug flow volume fraction,

(7) $$V_{P}/V=\left(\theta_{\min}+\theta_{\mathrm{peak}}\right)/2$$

Mixed flow volume fraction,

(8) $$V_{m}/V=1-V_{d}/V-V_{p}/V$$

where *τ* is the theoretical residence time, *θ*_min_ is the dimensionless time of minimum concentration at the tundish outlet, *θ*_peak_ is the dimensionless time of peak concentration at the tundish outlet. 

Another common RTD expression is the cumulative distribution function F(*t*), i.e., the F-curve. F-curve is a fraction of the liquid that has a residence time less than time (*t*) and can be obtained by making a continuous addition of tracers at the inlet. The concentration of tracers in the outlet stream is F-curve. In this study, F-curve was analyzed to evaluate an intermixing time exists between the value 0.2 and 0.8 of the dimensionless concentration of the tracer.

#### 2.1.3. Geometry, Mesh and Boundary Conditions 

**Figure 1:**
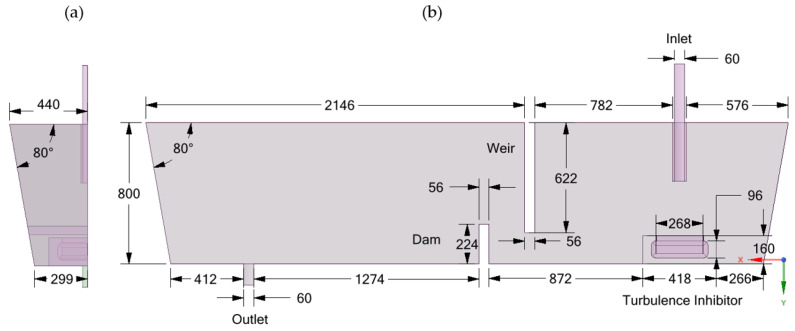
Dimensions of a single-strand tundish with flow control devices (dam, weir and turbulence inhibitor); (**a**) side view and (**b**) front view (unit: mm).

A single-strand tundish (14 tons) with a submerged inlet, an outlet, a weir, a dam and a turbulence inhibitor was under investigation in the current work. The geometric dimensions of the tundish are illustrated in Figure 1. The molten steel flow rate remained unchanged to keep a constant steel bath level in the tundish.

The volume mesh was generated in Star-CCM + V13 with the option of trimmer and prism layer. Three prism layers were generated next to all the walls. The surface mesh was generated first. Then, the volume mesh was built based on the surface mesh by adjusting the growth rate and the biggest mesh size. A base mesh size of 0.003 m and 0.006 m was used for the water model and prototype, respectively. The average *y +* value in the first layer of the mesh near the wall was 2. A half tundish model was simulated through its symmetry plane in order to save time on CFD calculations. It is a common approach for the tundish simulation when the Reynolds averaged Navier–Stokes (RANS) turbulence model is applied. However, it should be noted that a full-scale tundish model is recommended when the local 3D-transient phenomena are of interest to study (for example jet flow) or a large eddy simulation (LES) turbulence model is applied. The final CFD model possessed a total of 1.5 million trimmer cells in the computing domain. 

No-slip conditions were applied on all solid surfaces for the liquid steel phase. A constant mass flow was used at the inlet. At the outlet of tundish, the outflow boundary condition was applied. A wall function was used to bridge the viscous sub-layer and to provide the near-wall boundary conditions for the average flow and the turbulence transport equations.

The heat losses of the water model were ignored. In the simulation of the tundish prototype, the heat losses through the side and bottom walls were set to be 2.5 kW/m^2^. Three different heat losses through the top surface were studied, which were set to be 7.5, 15 and 30 kW/m^2^, respectively.

Zero mass flux was applied at walls and free surface for the passive scalar equation. At *t* = 0–2 s the mass fraction of tracer at the inlet was set to be equal to 1. When *t* > 2 s it was given as zero. The concentration of the tracer at the outlet was monitored from *t* = 0 to 2000 s and the RTD curves were obtained from the numerical calculation. A summary of input parameters and boundary conditions used for computational fluid dynamics simulations is given in Table 2.

#### 2.1.4. Solution Procedure

The discretized equations were solved in a segregated manner with the semi-implicit method for the pressure-linked equations (SIMPLE) algorithm. The second-order upwind scheme was used to calculate the convective flux in the momentum equations. The solution was judged to be converged when the residuals of all flow variables were less than 1 × 10^−4^, together with the stability of the velocity, the temperature and the turbulence at the key monitored points. The transient flow fields and temperature distribution were calculated for the non-isothermal water model with the variations of inlet temperature. The steady state flow fields and temperature distribution were calculated for the prototype with consideration of the heat losses in tundish. The under-relaxation parameters of flow calculations for the pressure, the velocity and the turbulence were 0.3, 0.7 and 0.8, respectively. To calculate the RTD curves in the prototype, the flow fields were first calculated in a steady state. Then, the transient calculations were performed to solve the passive scalar equations.

### 2.2. Non-Isothermal Water Model 

**Figure 2:**
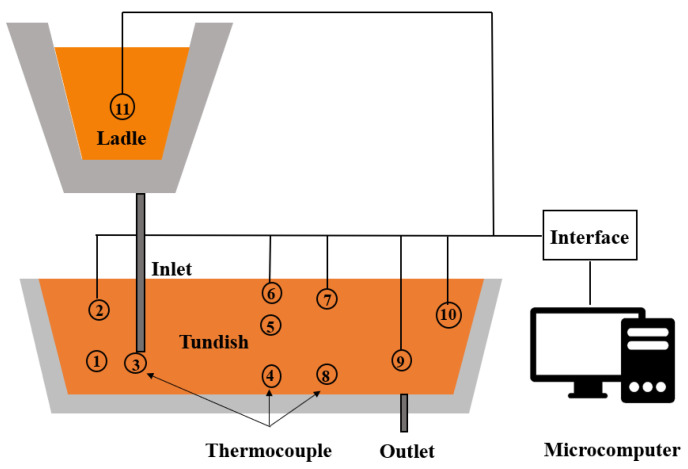
Schematic diagram of the experimental setup for physical modelling.

The experimental equipment of a non-isothermal water model is shown in Figure 2. The water model was constructed of plexiglass with the geometric scale ratio λ of 1:2. 

Dynamic similarity is determined by requiring the Froude number in the water model, which is equivalent to that in the prototype, as shown in Equation (9).

(*Fr*)_m_ = (*Fr*)_p_ (9)

where m stands for water model and p is the prototype of the tundish. The Froude number, *Fr*, is defined as Equation (10)

*Fr* = *u*^2^/*gL* (10)

Then, liquid velocity, volumetric flow rate and time ratio between the model and the prototype are described as a function of geometric scale ratio, according to the Froude similarity. Parameters of the water model are obtained by these equations, which are listed in Table 2.

A heater was attached to the upper water tank to keep the desired temperature difference between the upper tank and the water model. The dimensionless number *Gr/Re*^2^ was used as the thermal similarity criteria, defined in Equation (11). In the water model experiment, temperature change (ΔT) is defined as the difference between the inlet temperature and the bath temperature.

(11) $$\frac{G_{r}}{{R_{e}}^{2}}=\frac{buoyancy\;force}{Inertial\;force}=\frac{gl^{3}\beta \Delta T/{\left(\mu /\rho \right)}^{2}}{{\left(\rho ul/\mu \right)}^{2}}=\frac{g\beta \Delta Tl}{u^{2}}$$

The dimensionless number *Gr/Re*^2^ can be used to evaluate the convection pattern in the flow system, as follows:

*Gr/Re*^2^ << 1: inertial force dominates fluid flow, forced convection;

*Gr/Re*^2^≅ 1: both inertial and buoyancy force dominates fluid flow, mixed convection;

*Gr/Re*^2^ >> 1: buoyancy force dominates fluid flow, natural convection.

For the water model and the actual tundish prototype, the equivalence of temperature rises in two fluids can be defined as follows:

(*Gr/Re*^2^)_m_ = (*Gr/Re*^2^)_p_ (12)

The following relationship can be derived from Equations (11) and (12),

(13) $$\Delta T_{m}=\frac{\beta_{p}}{\beta_{m}}\Delta T_{p}=0.605\cdot \Delta T_{p}$$

where *β*_p_ is the volumetric thermal expansion of liquid steel, set to 0.000127 (1/K) [12]. *β*_m_ is the volumetric thermal expansion of water, set to 0.00021 (1/K) [34].

From Equation (13), it can be calculated that a step input of ΔT = 20 °C of the inlet stream in the water model corresponds to a step input of ΔT = 33 °C of liquid steel in the prototype.

The hotter or cooler incoming stream pours through the inlet and leaves through the outlet of the water model. In total, ten thermocouples were placed at the central plane in the water model for observing the temperature changes. One thermocouple (No.11) was used to control the inlet temperature. The locations of thermocouples are illustrated in Figure 2. The water model experiments were carried out under conditions with: (i) different flow control devices (FCD) and (ii) different inlet temperature. The detailed theoretical analysis, experimental procedure and test parameters can be found in previous work of the authors [35]. In the present study, the experimental data were revisited with the aim of validating the developed CFD model. 

## 3. Results and Discussions

### 3.1. Water Model

#### 3.1.1. Experimental Data of Hotter Inflow

**Figure 3:**
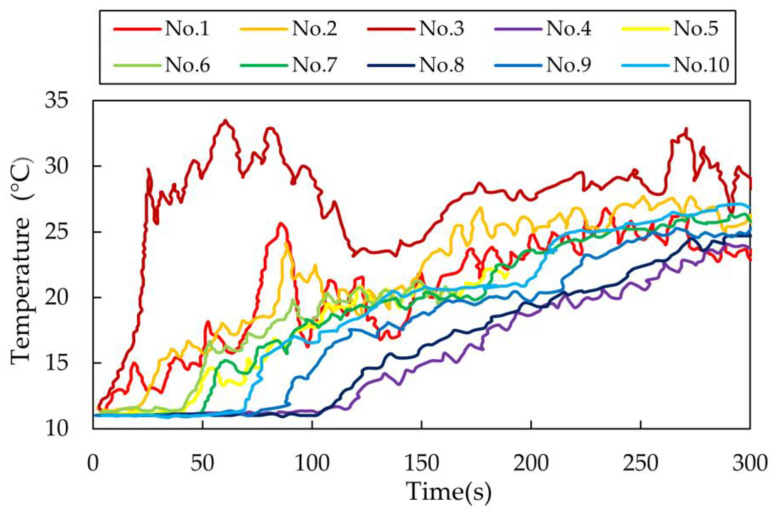
Temperature measurement at selected points in non-isothermal water model (Q = 2000 l/h, H = 0.4 m, ΔT = 30 °C).

**Figure 4:**
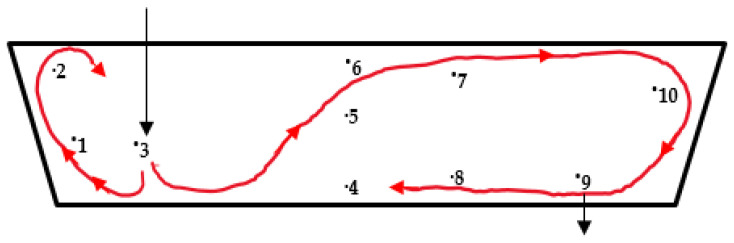
Thermal flow routes.

Figure 3 exhibits the plots of time vs. temperature for the selected 10 measurement points in the bare tundish under the conditions of Q = 2000 L/h, H = 0.4 m, ΔT = 30 °C. The initial response time of each thermocouple is listed in Table 3. It can be found that No.3 responded directly after pouring of the hotter incoming stream. The measured temperature of No.3 increased quickly. Then, No.1 and No.2 which were located near the inlet responded quickly as well due to the high turbulence flow in the inlet region. Afterwards, as listed in Table 3, the rise in temperature was subsequently detected from thermocouples No.5, 6, 7, 10, 9, 8 and 4, respectively. Thus, the thermal flow route inside the bare water model can be drawn in Figure 4. The hotter stream floated up and moved along the bath surface to the upright corner firstly, and then flowed along the right-side wall to the outlet. It can be observed in Figure 3 that the slower the thermocouples responded, the lower the temperature detected at the measurement point. The average measured temperature decreased gradually at the points of No.7, 10, 9, 8 and 4. The strong thermal buoyancy effect was also found in some previous water model studies [7,8,9,20]. 

#### 3.1.2. Validation of CFD Modelling 

Best practice guidelines (BPG) relevant to all numerical simulations are important to ensure accuracy and credibility of CFD predictions. The CFD model development in the present study followed the general guidelines from References [36,37,38]. The details of the validation of the CFD model, including mesh dependency study and comparison with the experimental data (RTD curves), can be found in previous works [39,40]. 

**Figure 5:**
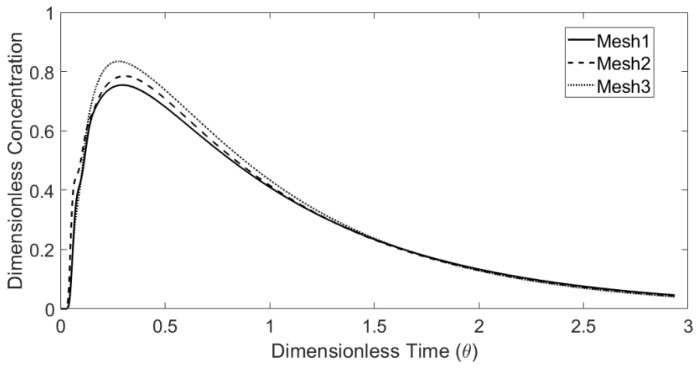
Comparison of calculated E-curves with different CFD mesh size (mesh size: Mesh1—0.002 m; Mesh2—0.003 m and Mesh3—0.004 m).

A mesh independency study was carried out to estimate a proper mesh density for the tundish water model is shown in Figure 5. The differences in the dead volume fraction (*V*_d_/*V*) of the three mesh sizes are less than 2%. An acceptable mesh independent solution was obtained based on the observations above. With the considerations of the computing load and the near wall resolution, the computations were carried out with a reference mesh size 0.003 m for the water model studies.

**Figure 6:**
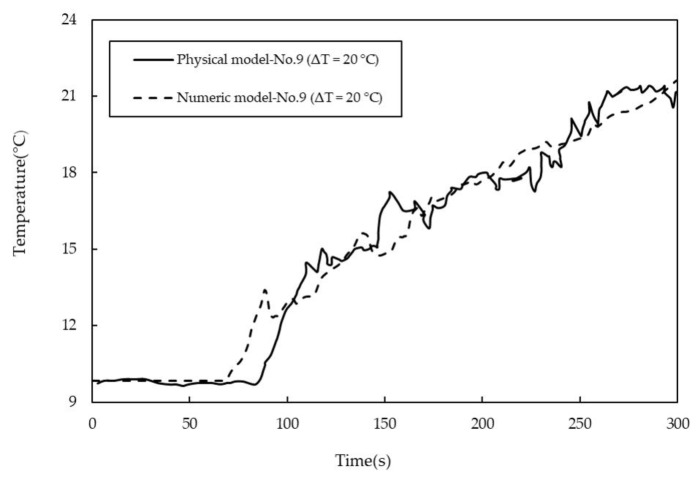
Comparison of temperature measurement at No.9 point in the non-isothermal water model (Q = 1500 L/h, H = 0.4 m, ΔT = 20 °C).

Figure 6 shows the measured and the predicted results of the temperature changes through time at point No.9 under the conditions of Q = 1500 L/h, H = 0.4 m and ΔT = 20 °C. The predicted temperature after pouring hotter water is in good agreement with the experimental results. However, a difference in the initial response time between the measurement and the prediction is observed. A possible reason is that the selected realizable k-ε model leads to an over prediction of the conductive heat transfer in the water model. 

#### 3.1.3. CFD Results of Hotter and Cooler Inflow (Bare Water Model) 

**Figure 7:**
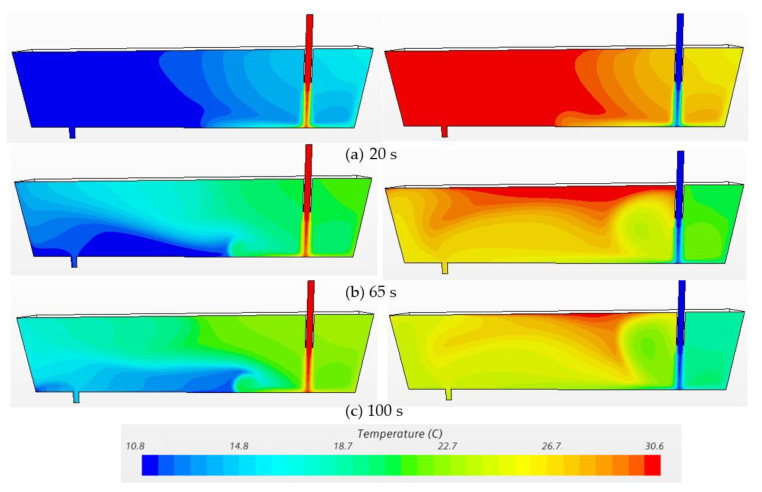
Temperature profiles at the symmetry plane under transient non-isothermal conditions in bare water model (Q = 2000 L/h, H = 0.4 m, left: ΔT = 20 °C, right: ΔT = −20 °C). (**a**) 20 s, (**b**) 65 s, (**c**) 100 s.

**Figure 8:**
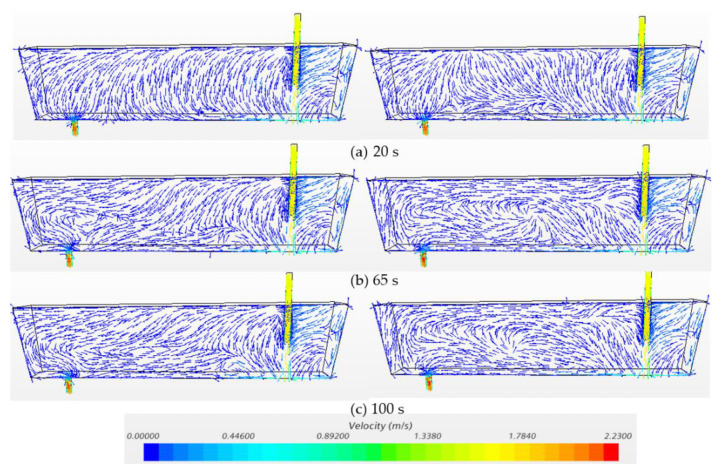
Velocity at symmetry plane under transient non-isothermal conditions in bare water model (Q = 2000 L/h, H = 0.4 m, left: ΔT = 20 °C, right: ΔT = −20 °C). (**a**) 20 s, (**b**) 65 s, (**c**) 100 s.

Figure 7 and Figure 8 exhibit the temperature profiles and flow vectors at the symmetry plane after pouring hotter (ΔT = 20 °C) and cooler (ΔT = −20 °C) inflow in the water model at t = 20 s, 65 s and 100 s, respectively. In order to clearly visualize the flow pattern, the vector length was not varied with respect to magnitude since the bath velocity was very small compared to the inlet velocity. The incoming flow jet quickly hits the bottom and forms an intense mixing near the inlet region (Figure 7a and Figure 8a). Then, the hot incoming stream floats upwards to the surface while the cold flow extends, spreading along the bottom (Figure 7b and Figure 8b) because the cold fluid is denser and heavier than the hot fluid. A counter-clockwise circulation is formed near the outlet region with the hotter inflow, while a clockwise recirculation is formed with the cooler inflow (Figure 7c and Figure 8c). Thermal stratification was observed in the water model due to natural convection (Figure 7c and Figure 8c). The characteristics of the hotter inflow lead to a strong surface flow and a longer residence time of fluid. On the other hand, the cooler incoming stream flows directly towards the outlet along the bottom, which shortens the residence time of fluids in the vessel. 

#### 3.1.4. CFD Results of Hotter and Cooler Inflow (Water Model with FCD)

**Figure 9:**
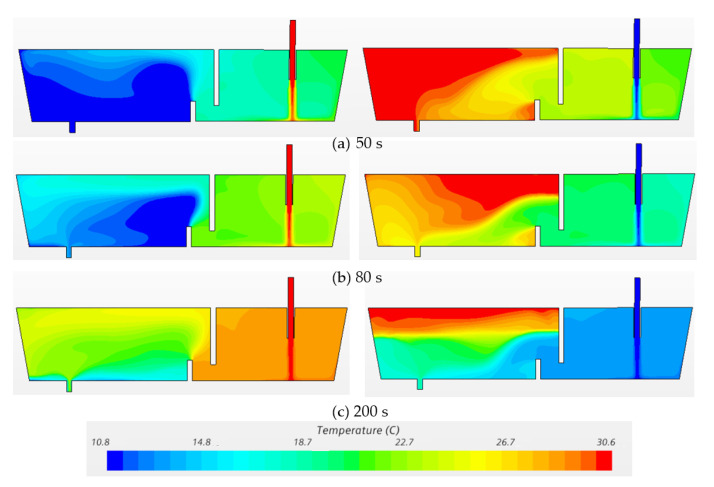
Temperature profiles at symmetry plane under transient non-isothermal conditions in water model with FCD (Q = 2000 L/h, H = 0.4 m, left: ΔT = 20 °C, right: ΔT = −20 °C). (**a**) 20 s, (**b**) 80 s, (**c**) 200 s.

**Figure 10:**
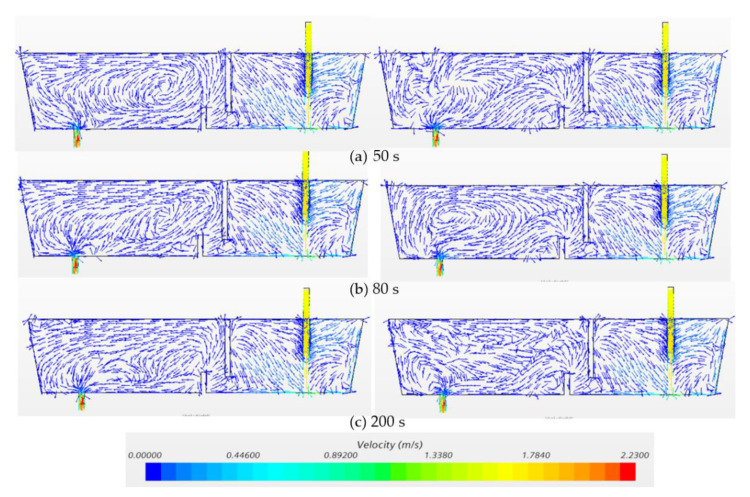
Velocity at symmetry plane under transient non-isothermal conditions in water model with FCD (Q = 2000 l/h, H = 0.4 m, left: ΔT = 20 °C, right: ΔT = −20 °C). (**a**) 20 s, (**b**) 80 s, (**c**) 200 s.

Figure 9 and Figure 10 show the temperature profiles and velocity vectors on the symmetry plane after pouring the hotter (ΔT = 20 °C) and cooler (ΔT = −20 °C) inflow in the water model with flow control devices (dam and weir) at t= 50 s, 80 s and 200 s, respectively. In comparison with the hotter inflow in Figure 7, the installation of the weir and dam strengthens the natural convection which therefore reduces the floating-up time of hot stream in the outlet chamber. In terms of the cooler inflow, the dam reorients the stream flowing upwards. However, the cold stream flows downwards just after it runs over the dam. A strong thermal stratification is observed in the vessel (Figure 9c). The hot liquid remains on the top of the outlet chamber which represents a fraction of dead zone in the vessel. Thus, the existence of weir and dam could not control the flow as expected with the cooler inflow. 

### 3.2. Prototype 

#### 3.2.1. Tundish Configuration—Weir and Dam 

**Figure 11:**
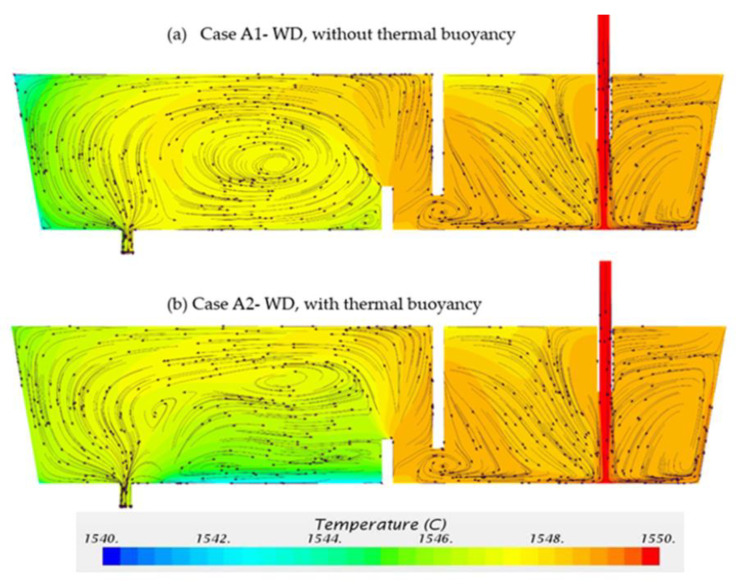
Temperature and flow pattern in (**a**) Case A1—without thermal buoyancy and (**b**) Case A2—with thermal buoyancy.

Two cases were calculated for the tundish configurations with weir and dam (WD): (i) Case A1—without thermal buoyancy, (ii) Case A2—with thermal buoyancy. Figure 11 shows the predicted flow pattern and temperature distributions on the symmetry plane after pouring the molten steel from ladle. The flow patterns in the entry zone have similar characteristics for the two cases. The entering liquid flows down to the bottom and spreads rapidly. The stream moves along the sidewall, then flows back to the incoming jet and forms counter flows near the inlet region. The turbulence zone caused by the incoming stream is confined within the region near the inlet owing to the presence of the weir. The flow moves underneath the weir and downstream towards the outlet chamber controlled by the dam. In the outlet chamber, a big counterclockwise circulation loop is observed in Case A1, without considering the thermal buoyancy. The circulation loop is squeezed in Case A2 due to the strong horizontal flow pattern caused by the thermal buoyancy. The similar thermal buoyancy effect in prototype tundish was also reported in some previous studies [6,11,22,24].

Comparing the temperature contours in Figure 11a,b, the temperature distributions are quite similar in the inlet chamber, however, they are obviously different in the outlet chamber. In Case A1 the lowest temperature is located in the upper-corner near the left-side wall due to the high heat loss and low velocity in that region. In Case A2 the lowest temperature is located near the bottom between the weir and the outlet. Due to high surface heat loss, thermal buoyancy drives the main stream towards the top surface, then flows along the top surface and left-side wall. A part of steel flows along the left-side wall towards the outlet. A part of steel, with relative lower temperature, flows to the bottom of tundish due to the characteristics of three-dimensional flow. The average temperature at outlet for both cases is 1546.7 °C, which is 3.3 °C lower than the inlet temperature.

**Figure 12:**
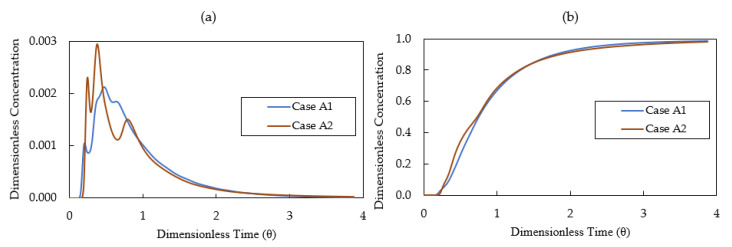
(**a**) E-curve and (**b**) F-curve for Case A1—without thermal buoyancy and Case A2—with thermal buoyancy.

Figure 12a displays the calculated E-curves for the two studied cases. The E-curve of Case A2 has a higher peak value and a higher variance compared with that of Case A1. The appearance of sharp peak in the E-curve indicates that short-circuiting flow phenomena existed in the flow system. In the region of short-circuiting, the fluids have no enough residence time and spaces to mix with the surrounding fluids, which is an undesirable feature in tundish operations. The analysis of RTD curves for Case A1 and Case A2 are listed in Table 4. The mean residence time for Case A1 and A2 is 469 s and 457 s, respectively. Case A2 has a higher dead volume fraction compared with Case A1. The comparison of Case A1 and Case A2 indicates that the mixing in non-isothermal conditions is lower than the mixing in isothermal conditions. This is because the warmer fluid floats to the free surface under non-isothermal conditions, leading to a thermal stratification in the bath.

Figure 12b illustrates the calculated results of the F-curves for Case A1 and Case A2. The F-curve provides useful data for the prediction of intermixing grade of casting product. The model assumes that an intermixing zone exists between the value 0.2 and 0.8 of the dimensionless concentration of the tracer. As listed in Table 4, the dimensionless concentration value of 0.2 at the tundish outlet, t (0.2), requires 228 s and 195 s for Case A1 and Case A2, respectively. This means that the new grade steel reaches the outlet faster when taking account of the thermal buoyancy. The intermixing time is 448 s and 478 s for Case A1 and Case A2, respectively. This indicates that the thermal buoyancy lengthens the intermixing time, thereby lowers the steel yields during the mixed grade casting process. 

#### 3.2.2. Tundish Configuration—Weir, Dam and Turbulence Inhibitor 

**Figure 13:**
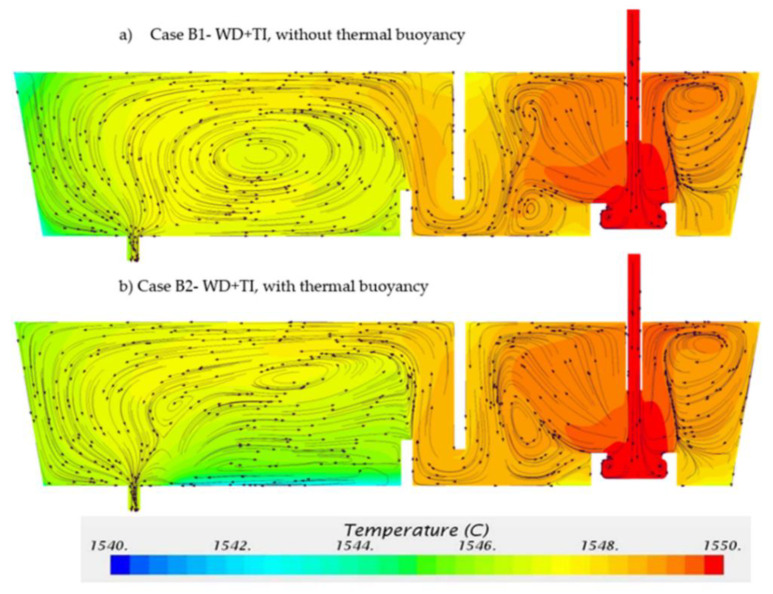
Temperature and flow pattern in (**a**) Case B1—without thermal buoyancy and (**b**) Case B2—with thermal buoyancy.

Two cases were calculated for the tundish configurations with weir, dam and turbulence inhibitor (WD+TI): (i) Case B1—without thermal buoyancy, (ii) Case B2—with thermal buoyancy. Figure 13 shows the predicted flow pattern and temperature distributions on the symmetry plane. When the tundish was equipped with a turbulence inhibitor, the entering flow reoriented towards the top surface and formed circulation loops in the inlet chamber. The appearance of turbulence inhibitor drove flow towards the surface with less turbulence, which can reduce the slag entrapment into the entering steam. The flow patterns in the outlet chamber show the similar behavior compared to Figure 11. The thermal buoyancy has a significant impact on the flow patterns.

As shown in Figure 13a,b, the temperature is more uniformly distributed in the inlet chamber in comparison with Figure 11. Thermal buoyancy changes the temperature distribution in the outlet chamber. In Case B1, the lowest temperature region is located at the upper-corner near the left-side wall. In Case B2, the lowest temperature region is located near the bottom between the weir and the outlet. The average temperature at outlet for both cases is 1546.7 °C which is 3.3 °C lower than in the inlet temperature, which remains the same as Case A1 and Case A2. 

**Figure 14:**
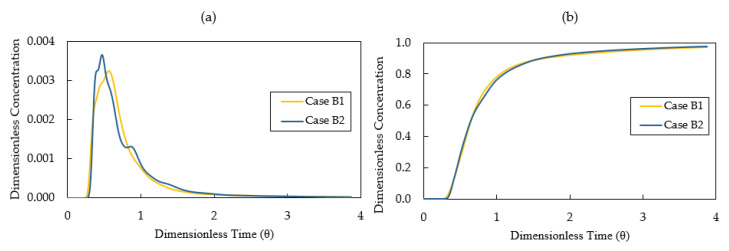
(**a**) E-curve and (**b**) F-curve for Case B1—without thermal buoyancy and Case B2—with thermal buoyancy.

Figure 14a displays the calculated E-curves for Case B1 and Case B2. E-curve of Case B2 has a higher peak value compared with that of Case B1. The difference between Case B1 and Case B2 becomes smaller compared with the difference between Case A1 and Case A2. This is mainly due to the strong mixing in the inlet chamber created by the turbulence inhibitor. The analyses of RTD curves for Case B1 and Case B2 are listed in Table 5. The mean residence time for Case B1 and Case B2 is 419 s and 425 s, which is shorter than Case A1 (469 s) and Case A2 (457 s). When the tundish was configured with only weir and dam (Case A1 and Case A2), the high momentum flow moves horizontally below the weir and downstream towards the outlet. The presence of a dam reorients the flow after the weir area in the outlet chamber. It drives the flow vertically upward to the top surface, which prolongs the residence time of molten steel. When the tundish was configured with weir, dam and turbulence inhibitor (Case B1 and Case B2), the horizontal momentum declines when the flow hits the dam. Consequently, the upward momentum decreases after the flow leaves the dam area. This shortens the flow path in the tundish, leading to a reduction of mean residence time and an expansion of dead volume fraction.

As a design criterion, the optimal tundish would rather have a big plug flow volume fraction and a small dead volume fraction [41]. As listed in Table 5, the plug flow volume fraction is bigger for both cases (Case B1, 25%; Case B2, 28%) compared to the results in Table 4 (Case A1, 14%; Case A2, 17%) which is the configuration without turbulence inhibitor. Meanwhile, the dead volume fraction is also bigger for both cases (Case B1, 19%; Case B2, 18%) in comparison with the results of Table 4 (Case A1, 9%, Case A2, 11%). With the aim of reducing the dead volume fraction, the gas bubbling technique or the baffle with deflector holes can be considered for the design improvement when the turbulence inhibitor is equipped in the tundish. 

Figure 14b illustrates the calculated results of the F-curves for Case B1 and Case B2. As listed in Table 5, the values of t (0.2) are similar for Case B1 and Case B2, 236 s and 233 s, respectively. Case B2 (334 s) has a longer intermixing time than Case B1 (307 s) due to the effect of thermal buoyancy. 

#### 3.2.3. Influence of Surface Heat Loss

**Figure 15:**
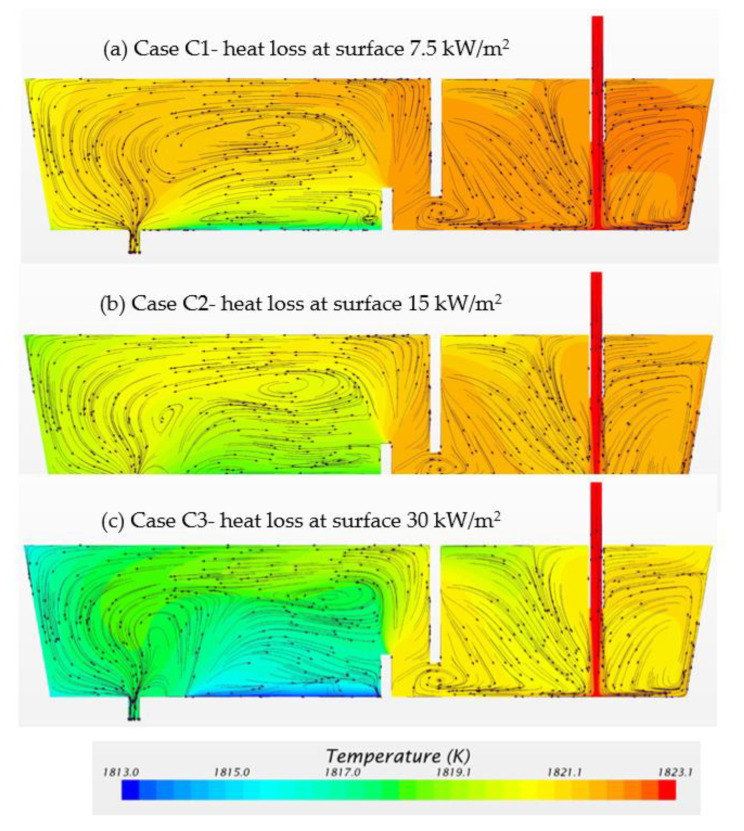
Temperature and flow movement for three cases with different surface heat losses: (**a**) Case C1: 7.5 kW/m^2^; (**b**) Case C2: 15 kW/m^2^; (**c**) Case C3: 30 kW/m^2.^

Three cases are calculated for the tundish configured with weir and dam, based on different heat losses through top bath surface: (i) Case C1, 7.5 kW/m^2^; (ii) Case C2, 15 kW/m^2^ and iii) Case C3, 30 kW/m^2^. Figure 15 presents the predicted flow pattern and temperature distributions on the symmetry plane. The flow patterns in the inlet chamber are quite similar for all three cases. In the outlet chamber, the flow patterns are different for the three cases. Case C3 shows the highest temperature gradient due to the highest heat loss. It is of interest to notice that there is a lowest temperature region near the bottom of tundish in Case C3 which can be termed as a dead zone due to the low velocities of the fluid. CFD results reveal that the flow surrounding the lowest temperature region becomes more complex due to the natural convection caused by the high temperature gradient. 

**Figure 16:**
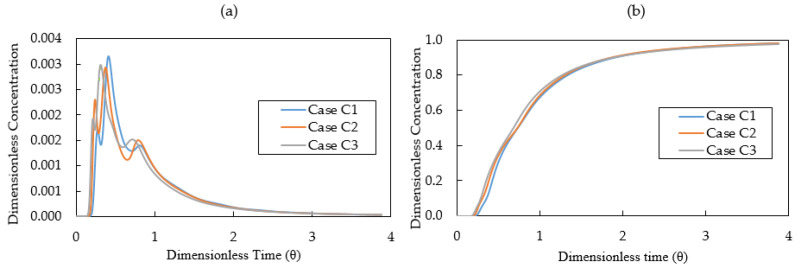
(**a**) E-curve and (**b**) F-curve for different heat flux at top surface (Case C1: 7.5 kW/m^2^, Case C2: 15 kW/m^2^, Case C3: 30 kW/m^2^).

Figure 16a displays the calculated E-curves for Case C1, Case C2 and Case C3. The three E-curves have similar shapes indicating the similar flow patterns. The analysis results of E-curves for Case C1, Case C2 and Case C3 are listed in Table 6. When increasing the surface heat loss, it brings a reduction of mean residence time, an expansion of dead volume fraction and a decrease of plug volume fraction. The CFD results reveal that the surface heat loss influences not only the superheat of molten steel, but also the flow patterns in the tundish. It is important to decrease the surface heat loss with the aim of improving the tundish performance.

Figure 16b illustrates the calculated results of the F-curves for Case C1, Case C2 and Case C3. As shown in Table 6, the value of t (0.2) decreases when increasing the surface heat loss. This is caused by a strong natural convection flow near the surface and walls when the surface heat loss is large. Meanwhile, the surface heat loss has a minor effect on the intermixing time.

## 4. Conclusions

A CFD model has been utilized to predict fluid flow and residence-time distribution in a single-strand tundish. The main findings of the numerical investigation are summarized as follows.

1. When pouring the hotter or cooler water into the water model without flow control devices, the numerical and experimental results clearly show a thermal-driven flow and a strong thermal stratification in the bath.
2. When pouring the cooler water into the water model with weir and dam, CFD results show that the main stream flows downwards after running over the dam, then moves along the bottom toward the outlet. The dam is not effective to control the flow when the inlet temperature is lower than the bath temperature.
3. The thermal-driven flow is observed in the prototype furnished with weir and dam when considering the heat losses and keeping the inlet temperature unchanged. In addition, RTD analysis shows that the degree of mixing in the tundish is lower when taking heat losses into account.
4. The thermal-driven flow is also observed in the tundish furnished with weir, dam and turbulence inhibitor. The presence of turbulence inhibitor increases the plug volume fraction. However, it also leads to a growth of the dead volume fraction due to the impaired function of the dam.
5. When increasing the surface heat loss, it results in an increase of the dead volume fraction and a decrease of the plug volume fraction. The surface heat loss influences not only the superheat of molten steel, but also the flow patterns in the tundish.

To sum up, thermal buoyancy has a significant effect on flow pattern and residence-time distribution in the tundish. A better control of thermal status is important to improve tundish performance. The validation of mathematical model by comparison with the plant trial data is highly desirable. The transient phenomena during an entire ladle changeover operation need to be considered in future work. Moreover, a conjugated heat transfer model including refractory layer and slag layer needs to be further developed with the aim of a more accurate prediction of heat losses in tundish. 

## Figures and Tables

**Table 1:** Summary of mathematical modelling investigations in a tundish under non-isothermal conditions.

Reference	Model ^1^	Code	Design			Numeric Model ^4^	Heat Flux(kW/m^2^) ^5^		Cond. ^6^	Thermal Convection ^2^	Study Focus ^7^
			Str. ^2^	Flu. ^2^	FCD ^3^		Top	Wall (B/L/T)			
Joo (1993) [6]	N	METFLO	1	S	W, D	-	-	2.6		Boussinesq	TP, TC, IRR, S,
Barreto (1996) [7]	N, P	-	1	S/W	-	E/k-ε	15	-/3.8/3.2	41/0.597	Density change	RTD, TC, V
Damle (1996) [8]	N, P	FORTRAN	2	S/W	-	E/k-ε	0	0	-	Density change	RTD, FR
Vargas-Zamora (2003) [9]	N, P	-	1	W	TI, D	E/k-ε/Inc.	0	0	-	Density change	CIT, FP, BF, TM, TOI, TD
Alizadeh (2008) [10]	N, P	-	2	W	D	Inc.	0	0	-	Mixed model	CIT
Braun (2010) [11]	N, P	FLUENT	2	S/W	SR	E/k-ε	32	10.91	-	Boussinesq	FP
Chattopadhyay (2012) [12]	N, P	FLUENT	4	S/W	TI	E/k-ε/Inc.	0	0	-	Boussinesq	IRR, TC, TD, TM, FP
Qu (2012) [13]	N	-	1	S	TI, W, D, SR	E/k-ε	15	1.4/3.2/3.8	41	Density change	TM, TP, FP, IT
Singh (2012) [14]	N	FLUENT	1	S	IW, TI, B, D	E/k-ε	21	1.96/4.48/5.32	-	Boussinesq	CIT, TP, FP, TC
Sun (2012) [15]	N, P	-	1	S/W	TI, W, D	E/k-ε	15	1.43/3.8/3.2	28	Density change	FP, V, RTD
Ling (2013) [16]	N	-	2	S	D, W, SR	E/k-ε/Inc	15	1.4/3.2/3.8	-	-	IRR, IND, IS
Hamid (2013) [17]	N, P	-	4	S/W	TI	-	-	-	-	Density change	SM, TM, RTD
Wang (2014) [18]	N, P	CFX	1	S/W	I	E/k-ε	15	1.8/5.2/4.6	41	Density change (S)	EMF, V, TP, TD
Tripathi (2015) [19]	N	FLUENT	1	S	TI	E/k-ε	(Tuning with plant)		41	-	CIT, FP, IND
Chatterjee (2017) [20,21]	N, P	FLUENT	4	S/W	IP	E/k-ε/Inc.	75	2.5	41/0.6	Boussinesq	CIT, TOI, FP, TD, TP, IRR
Cwudziński (2017) [22]	N, P	FLUENT	1	S/W	D, IW	E/k-ε	15	2.6/1.75/1.75	41	Density change	CIT, TC, HF
Yue (2017) [23]	N, P	CFX	7	S	-	E/k-ε	15	1.8/4.6/5	37	Density change	IH, TM, FP, V
Tang (2018) [24]	N, P	FLUENT	7	S/W	TI, C	E/k-ε	15	1.8/4/4	41	Density change	TC, TD, TP, FP, CI, IS, IRR
Ramirez (2018) [25]	N	FLUENT	5	S	IP, D	VOF/k-ε	15	1.4/3.2/3.8	32.7	Density change	BH, TP, IS, TC, IRR
Xing (2019) [26]	N	FLUENT	1	S	C	E/k-ε	0	0	-	Density change	TM, FP, RTD, TI, IRR, HP
Agarwal (2019) [27]	N	FLUENT	6	S	TI	-	18	1.8	35	Boussinesq	FP, TP, V, TM, RTD, TT

^1^ N: numerical model; P: physical model; ^2^ Str.: Strand; Flu.: Fluid; W: water; S: steel; ^3^ FCD: flow control device; B: baffle; C: channel; D: dam; I: induction; IP: impact pad; IW: Inclined wall; M: magnetic; SR: stop rod; TI: Turbulence inhibitor; W; weir; ^4^ E: Eulerian; Inc.: inclusion; ^5^ B: bottom; L: longitudinal; T: transitional; ^6^ Cond.: thermal conductivity (W m^−1^ K^−1^); ^7^ BF: buoyancy force; CI: channel inclination; CIT: comparison with iso-thermal; EMF: electromagnetic field; FP: flow pattern; FR: flow rate; FP: fluid property (liquid height, etc.); IH: induction heating. IND: Inclusion number density; IRR: inclusion removal rate; IS: inclusion size; IT: initial temperature; HF: heat flux; HP: heating power; S: state; SM: slag movement; TC: tundish configuration; TD: tracer dispersion; TOI: trajectories of inclusions; TP: temperature profile; TM: temperature measurement; TT: transition tonnage; V: velocity.

**Table 2:** Input parameters and boundary conditions used for computational fluid dynamics (CFD) simulations.

Parameter	Water Model	Prototype
Density	997 kg/m^3^	7020 kg/m^3^
Viscosity	0.00089 Pa·s	0.0062 Pa·s
Reference pressure	101,325 Pa	101,325 Pa
Heat capacity	4200 J/kg·K	760 J/kg·K
Thermal conductivity	0.6 W/m·K	41 W/m·K
Thermal expansion coefficient	0.00021 1/K	0.000127 1/K
Liquid level	0.4 m	0.8 m
Inlet (flow)	2400 L/h	14,000 L/h
Inlet (temperature, ΔT)	ΔT = ±20 °C	T = 1550 °C, ΔT = 0 °C
Wall (flow)	No slip	No slip
Surface (flow)	Free slip	Free slip
Wall (heat loss)	0 kW/m^2^	2.5 kW/m^2^
Surface (heat loss)	0 kW/m^2^	7.5,15,30 kW/m^2^
Tracer inlet (E-curve)	1 (t ≤ 0–2 s), 0 (t > 2 s)	1 (t ≤ 0–2 s), 0 (t > 2 s)
Tracer inlet (F-curve)	1	1

**Table 3:** Breakthrough time of different thermocouples.

**Time (s)**	5	10	30	45	45	50	70	80	100	120
**Probe**	3	1	2	5	6	7	10	9	8	4

**Table 4:** RTD analysis for Case A1 and Case A2.

Case	Mean RT s	t(min) s	t(max) s	t(0.2) s	t(0.8) s	Intermixing Time, s	*V*_d_/*V* %	*V*_p_/*V* %	*V*_m_/*V* %
Case A1	469	70	243	228	676	448	9	14	77
Case A2	457	88	194	195	673	478	11	17	72

**Table 5:** RTD analysis for Case B1 and Case B2.

Case	Mean RT s	t(min) s	t(max) s	t(0.2) s	t(0.8) s	Intermixing Time, s	*V*_d_/*V* %	*V*_p_/*V* %	*V*_m_/*V* %
Case B1	419	129	291	236	543	307	19	25	56
Case B2	425	143	243	233	567	334	18	28	55

**Table 6:** Computational RTD parameters and the volume fraction of flow for Case C1, C2 and C3.

Case	Mean RT s	t(min) s	t(max) s	t(0.2) s	t(0.8) s	Intermixing Time, s	*V*_d_/*V* %	*V*_p_/*V* %	***V*** **_m_** **/*V* %**
Case C1	470	99	214	217	686	469	9	19	72
Case C2	457	88	194	195	673	478	11	17	72
Case C3	438	77	162	179	653	474	15	15	70

## Data Availability

The data presented in this study are available on request from the corresponding author.

## Nomenclature

*C(t)*	Tracer concentration at time *t*	*V*	Volume of tundish
*C_p_*	Heat capacity	*V_p_*	Plug flow volume
*D_eff_*	Effective diffusivity	*V_m_*	Mixed flow volume
*E(t)*	Residence-time distribution	*V_d_*	Dead zone volume
*E(θ)*	Dimensionless residence-time distribution	*x_j_*	Cartesian coordinates
*Fr*	Froude number	*β_p_*	volumetric thermal expansion of liquid steel
*G_k_*	Generation term of turbulent kinetic energy	*β_m_*	volumetric thermal expansion of water
*Gr*	Grashof number	*ε*	Turbulent energy dissipation rate
*H*	Bath height	*µ*	Molecular viscosity
*k*	Turbulent kinetic energy	*µ_t_*	turbulent viscosity
*L*	Length	*ρ*	Density
*Pr*	Turbulent Prandtl number	*τ*	Theoretical residence time
*Q*	Volumetric flow rate	*θ*	Dimensionless time
*Re*	Reynolds number	*θ_min_*	breakthrough time
*S_T_*	Source term of energy equation	*θ_peak_*	Peak dimensionless time
*t*	Time	*σ_k_*	turbulent Prandtl numbers for *k*
*u*	Velocity	*σ_ε_*	turbulent Prandtl numbers for *ε*
*υ*	Kinematic viscosity	*Y_M_*	Dilatation dissipation term
